# Data mining on identifying diagnosis and prognosis biomarkers in head and neck squamous carcinoma

**DOI:** 10.1038/s41598-023-37216-8

**Published:** 2023-06-20

**Authors:** Guoyuan Ju, Zhangyu Yao, Yanbin Zhao, Xiaotong Zhao, Fangzhou Liu

**Affiliations:** 1 The Affiliated Cancer Hospital of Nanjing Medical University, Nanjing, 210029 China; 2 Department of Head and Neck Surgery, Jiangsu Cancer Hospital and Jiangsu Institute of Cancer Research and, The Affiliated Cancer Hospital of Nanjing Medical University, Nanjing, 210029 China; 3 Department of Otorhinolaryngology and Head and Neck Surgery, Affiliated Hospital of Xuzhou Medical University, Xuzhou, 221000 Jiangsu China

**Keywords:** Biological techniques, Cancer, Oncology

## Abstract

Head and neck squamous carcinoma (HNSC) induces high cancer-related death worldwide. The biomarker screening on diagnosis and prognosis is of great importance. This research is aimed to explore the specific diagnostic and prognostic biomarkers for HNSC through bioinformatics analysis. The mutation and dysregulation data were acquired from UCSC Xena and TCGA databases. The top ten genes with mutation frequency in HNSC were *TP53* (66%), *TTN* (35%), *FAT1* (21%), CDKN2A (20%), MUC16 (17%), CSMD3 (16%), PIK3CA (16%), NOTCH1 (16%), SYNE1 (15%), LRP1B (14%). A total of 1,060 DEGs were identified, with 396 up-regulated and 665 downregulated in HNSC patients. Patients with lower expression of *ACTN2* (*P* = 0.039, HR = 1.3), *MYH1* (*P* = 0.005, HR = 1.5), *MYH2* (*P* = 0.035, HR = 1.3), *MYH7* (*P* = 0.053, HR = 1.3), and *NEB* (*P* = 0.0043, HR = 1.5) exhibit longer overall survival time in HNSC patients. The main DEGs were further analyzed by pan-cancer expression and immune cell infiltration analyses. MYH1, MYH2, and MYH7 were dysregulated in the cancers. Compared with HNSC, their expression levels are lower in the other types of cancers. *MYH1*, *MYH2*, and *MYH7* were expected to be the specific diagnostic and prognostic molecular biomarkers of HNSC. All five DEGs have a significant positive correlation with CD4+T cells and macrophages.

## Introduction

Head and neck squamous carcinoma (HNSC) accounts for 95% of head and neck cancer (HNC) and is the sixth most common malignancy worldwide^1^. It contains neck, ear, nose, throat, and oral and maxillofacial tumors^2,3^. Human papillomavirus (HPV) infection is the most common causative factor for HNSC^4–6^. Although advanced treatments have been employed in HNSC treatment, the 5-year survival rate is less than 50% in stage III and IV HNSC^7^. Due to the difficulty in detecting HNSC at an early stage, the delay in detection and follow-up raises the risk of mortality. To control the mortality rate, biomarker identification in diagnosis, prognosis, and metastasis is very important, which might provide essential evidence for clinical treatment.

Machine learning based on public databases provides enormous value for somatic mutation and transcriptomes data mining^8^. The Cancer Genome Atlas (TCGA) datasets were generally used as databases containing various mutation and expression data of cancers. In recent years, machine learning and data mining have contributed to biomarker screening for early detection, diagnosis, drug application, metastases, and prognosis of various cancers^9–11^. For example, by employing four machine learning models, Leitheiser et al.^12^ predicted the primary sites of HNSC metastases based on DNA methylation. Rendleman et al.^13^ developed a machine learning method to improve the usability of the HNSC dataset for enhancing future oncological decisions and clinical applications. Jiang et al.^14^ provided potential predictive value for HNSC by employing public datasets. However, the data mining on the prognosis biomarker of HNSC is still inadequate.

Realizing the excellent application prospect of machine learning and the urgent need for reliable prognosticators for HNSC, we tried to identify the biomarkers related to diagnosis and prognosis in HNSC based on gene mutation and dysregulation. Moreover, we attempted to investigate the immune infiltration of DEGs in HNSC. This study may provide a specific set of molecular biomarkers for HNSC.

## Results

### Mutations in HNSC patients

**Figure 1:**
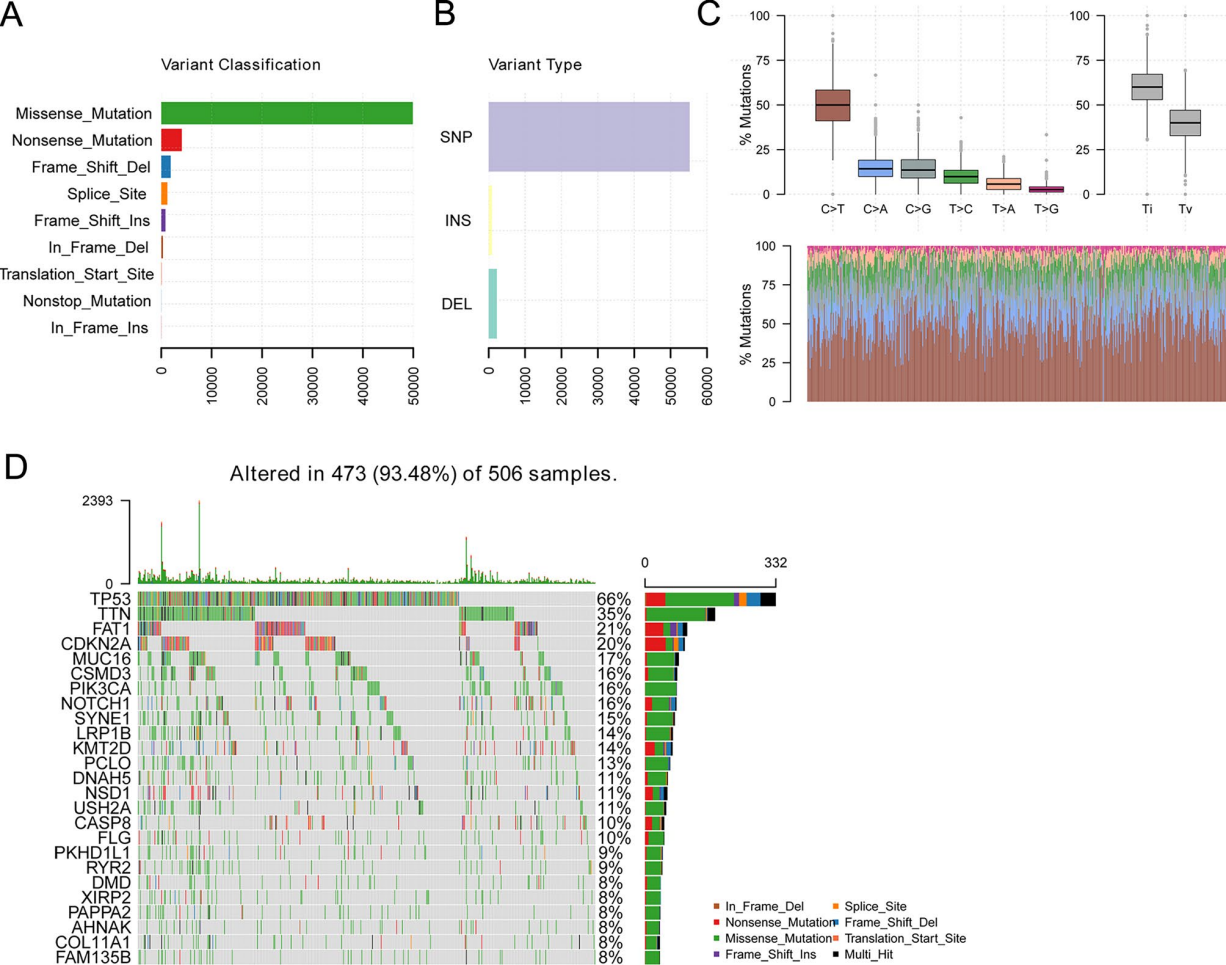
The landscape of mutation profiles in HNSC patients. (**A**,**B**) classification of mutation types according to different categories. (**C**) the frequency distribution of SNV mutation types. Categorizing SNPs as Transitions_vs_Transversions, the aggregated data can also be displayed as a boxplot showing the overall distribution of the six different transitions, and as a stacked bar chart showing the proportion of transitions in each sample. (**D**) showed Waterfall plot of the top 25 mutated genes in TCGA HNSCC cohort. The first part is the heatmap in the middle, where each row represents a gene and each column represents a sample, showing the distribution of different mutation types for each sample, and the second part is a stacked bar chart on the right, representing the different mutation types on each gene The frequency distribution of the loci, the third part is the stacked histogram above, which represents the frequency distribution of the loci of different mutation types in each sample.

The mutation profile of HNSC patients from the TCGA database was obtained. Figure 1A showed that the frequency distribution of different variant types was divided into seven according to the impact on protein-coding. We found that missense mutation accounts for the majority of the variant types. In these variant types, the number of SNPs is significantly more than that of INS and DEL (Fig. 1B). Besides, C > T transversion was the primary type of single nucleotide variants (SNV) in HNSC (Fig. 1C). We show genes with a mutation frequency of top25 in Fig. 1D. The top ten genes with a mutation frequency were TP53 (66%), TTN (35%), FAT1 (21%), CDKN2A (20%), MUC16 (17%), CSMD3 (16%), PIK3CA (16%), NOTCH1 (16%), SYNE1 (15%), LRP1B (14%).

### Differentially expression genes calculation

**Figure 2:**
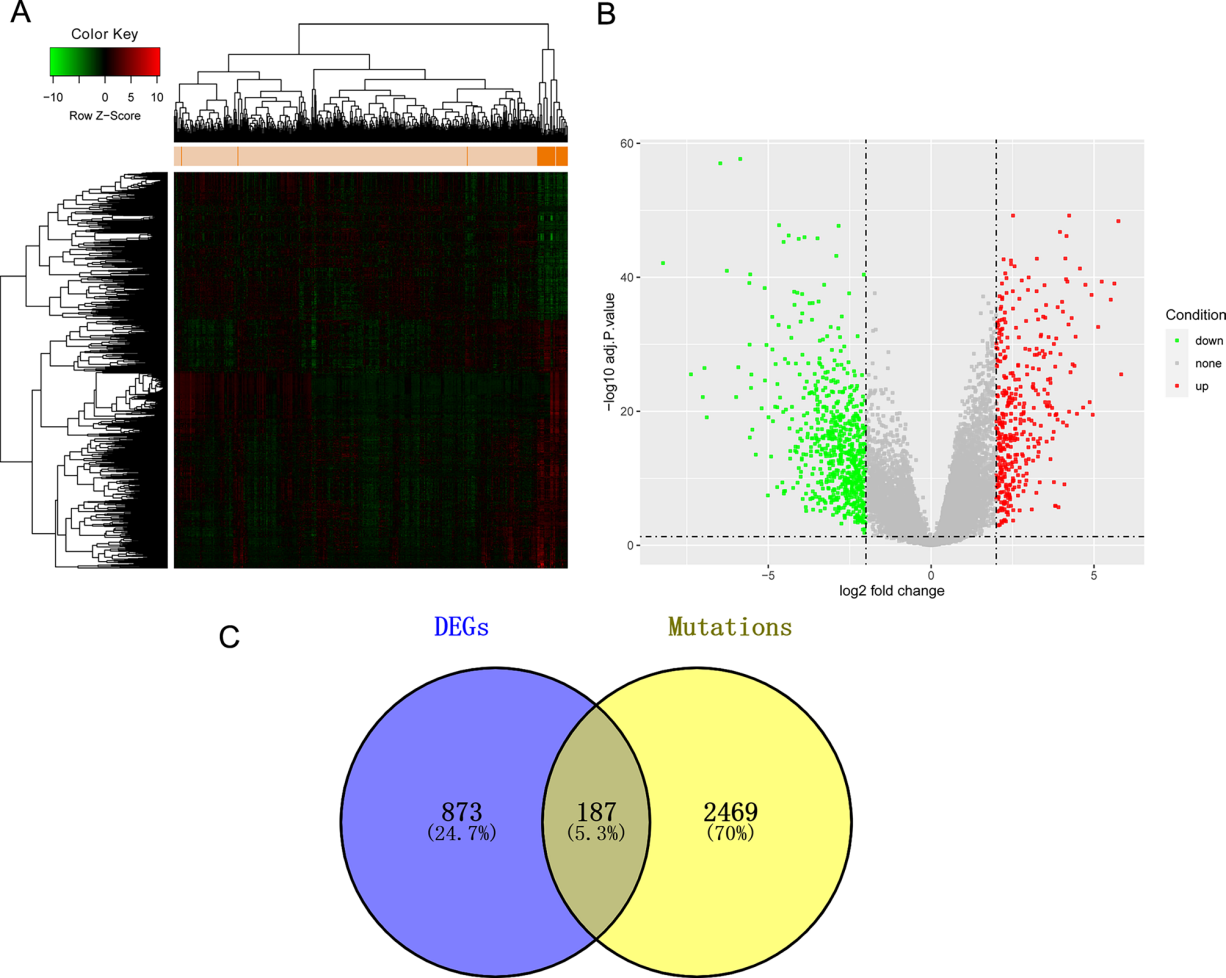
The DEGs identification. (**A**,**B**) exhibited the heatmap and volcano of DEGs. (**C**) the Venn chart showed the retained DEGs with more mutation frequency.

In these patients, the expression levels in two groups were compared, and a total of 1061 DEGs were identified, with 396 up-regulated and 665 downregulated (Fig. 2A,B). Then, we retained the DEGs with more than 1% mutation frequency. After the screening, 187 common DEGs were obtained (Fig. 2C), including 66 up-regulated and 121 down-regulated.

### PPI and key module analyses on DEGs

**Figure 3:**
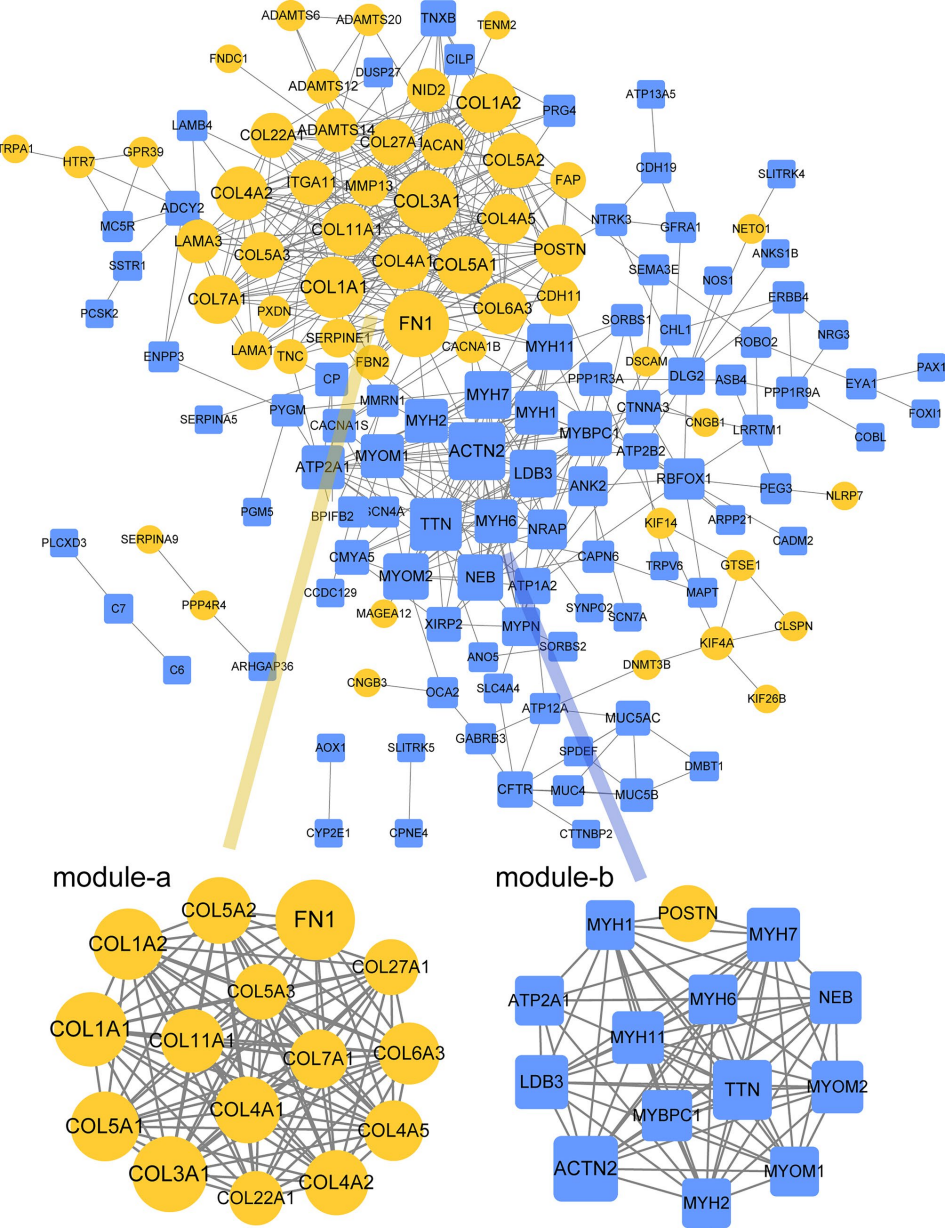
PPI and key module analyses on DEGs. The yellow circle is the down-regulated gene, the blue prism is the up-regulated gene, the node size is based on the degree value, the higher the degree value, the larger the node.

PPI analysis was performed on the common DEGs with more mutation frequency. The PPI network showed 144 nodes and 489 interaction pairs (Fig. 3). The DEGs with more than one connection with other DEGs were retained (Supplementary File 1). In addition, two sub-network modules were screened, with 15 nodes and 102 interaction pairs in module A (score = 14.571), and 14 nodes and 75 interaction pairs in module B (score = 11.538).

In addition, we listed the key DEGs in sub-network modules in Table 1.

**Table 1:** Key DEGs in sub-network modules.

Module A			Module B		
Nodes	Regulation	Degree	Nodes	Regulation	Degree
FN1	UP	31	**ACTN2**	DOWN	24
COL3A1	UP	28	TTN	DOWN	20
COL1A1	UP	27	POSTN	UP	18
COL1A2	UP	25	**MYH7**	DOWN	16
COL5A1	UP	24	LDB3	DOWN	16
COL5A2	UP	22	MYOM2	DOWN	15
COL4A1	UP	22	MYH11	DOWN	15
COL4A2	UP	21	**NEB**	DOWN	15
COL11A1	UP	20	MYBPC1	DOWN	14
COL6A3	UP	19	MYOM1	DOWN	13
COL4A5	UP	18	MYH6	DOWN	13
COL7A1	UP	17	**MYH1**	DOWN	13
COL5A3	UP	15	**MYH2**	DOWN	13
COL27A1	UP	15	ATP2A1	DOWN	13
COL22A1	UP	14			

DEG, differentially expressed genes.

### Function analysis on DEGs in sub-network modules

**Figure 4:**
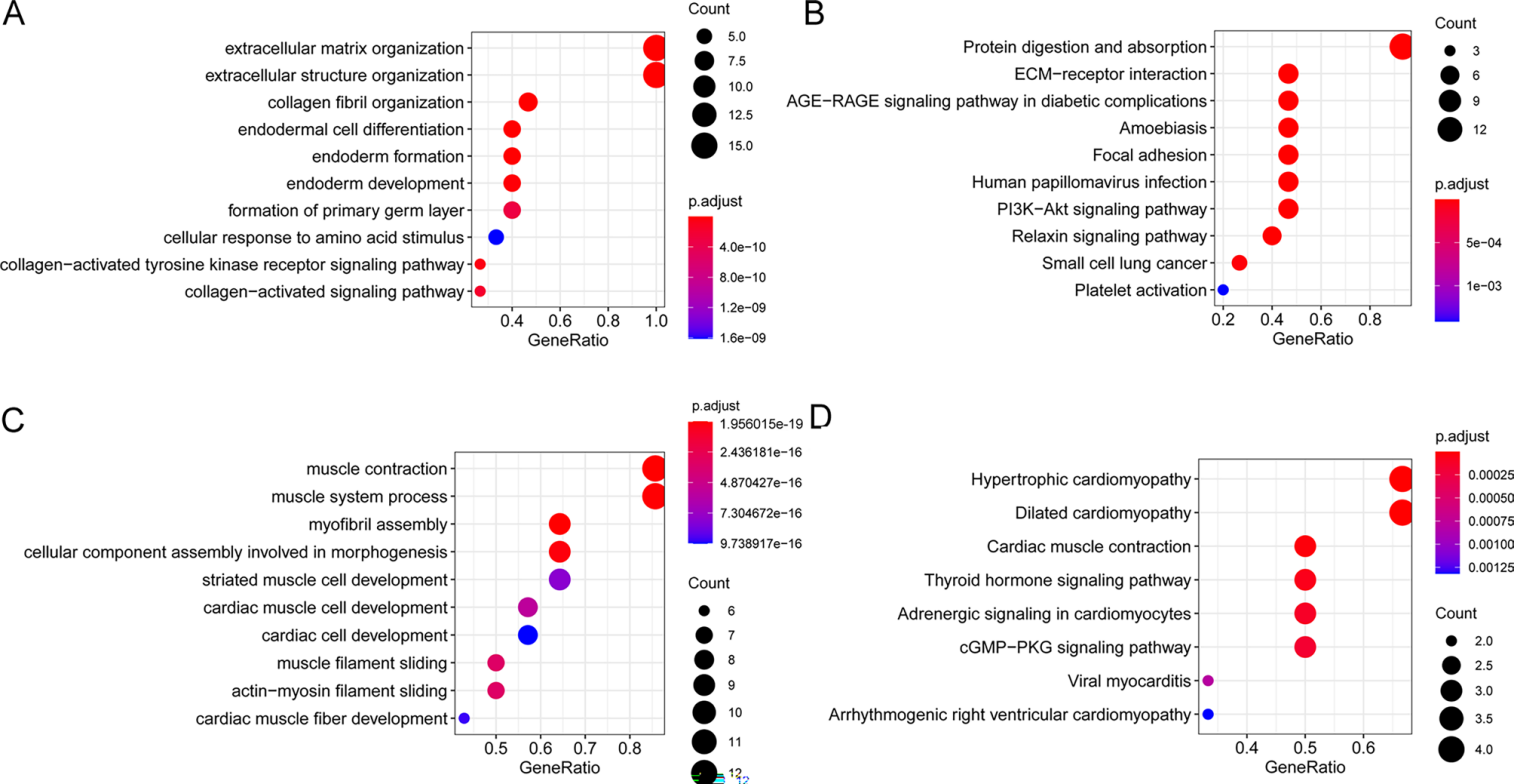
The GO-BP and KEGG analyses on DEGs in sub-network modules. (**A**) The GO-BP analysis on DEGs in sub-network module A. (**B**) The KEGG analysis on DEGs in sub-network module A. (**C**) The GO-BP analysis on DEGs in sub-network module B. (**D**) The KEGG analysis on DEGs in sub-network module B.

We further analyzed the function of DEGs by GO-BP and KEGG. We found that the DEGs in module A are mainly involved in GO-BP terms, including extracellular matrix organization, extracellular structure organization, collagen fibril organization, endodermal cell differentiation, endoderm formation, endoderm development, formation of the primary germ layer, cellular response to amino acid stimulus, collagen-activated tyrosine kinase receptor signaling pathway, and collagen-activated signaling pathway (Fig. 4A). They are also involved in KEGG pathways of protein digestion and absorption, ECM-receptor interaction, AGE-RAGE signaling pathway in diabetic complications, amoebiasis, focal adhesion, human papillomavirus infection, PI3K-Akt signaling pathway, Relaxin signaling pathway, small cell lung cancer, and platelet activation (Fig. 4B).

The DEGs in module B mainly participated in muscle contraction, muscle system process, myofibril assembly, cellular component assembly involved in morphogenesis, striated muscle cell development, cardiac muscle cell development, cardiac cell development, muscle filament sliding, actin-myosin filament sliding, and cardiac muscle fiber development (Fig. 4C). In addition, KEGG pathways of hypertrophic cardiomyopathy, dilated cardiomyopathy, cardiac muscle contraction, thyroid hormone signaling pathway, adrenergic signaling in cardiomyocytes, cGMP-PKG signaling pathway, viral myocarditis, and arrhythmogenic right ventricular cardiomyopathy were enriched (Fig. 4D).

### Key DEG expression validation, survival curve, and UALCAN Pan-Cancer analysis

**Figure 5:**
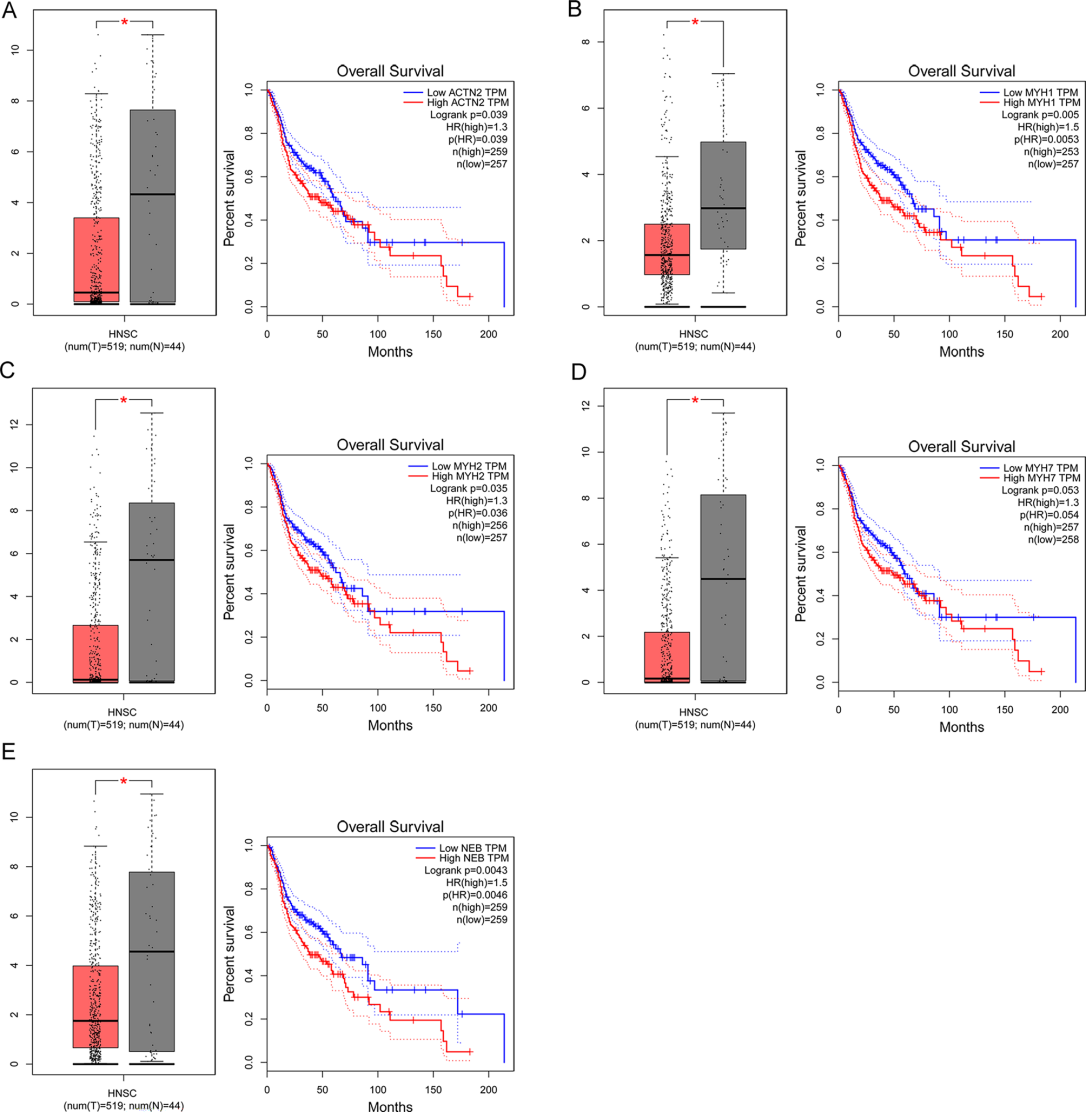
Key DEG expression validation and survival curve analyses. The expression levels and overall survival analysis of ACTN2, MYH1, MYH2, MYH7, and NEB by GEPIA.

Some of the key DEGs were validated by GEPIA. ACTN2, MYH1, MYH2, MYH7, and NEB were significantly downregulated in HNSC patients (*P* < 0.05; Fig. 5). Patients with lower expression of ACTN2 (*P* = 0.039, HR = 1.3), MYH1 (*P* = 0.005, HR = 1.5), MYH2 (*P* = 0.035, HR = 1.3), MYH7 (*P* = 0.053, HR = 1.3), and NEB (*P* = 0.0043, HR = 1.5) exhibit longer overall survival time. These results demonstrated that these DEGs might be essential for predicting the overall survival of HNSC.

**Figure 6:**
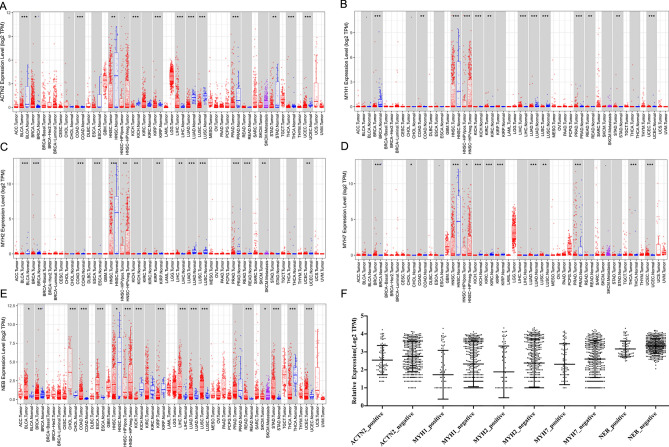
The expression of ACTN2, MYH1, MYH2, MYH7, and NEB in Pan-cancers. (**A**–**E**) represented the expression levels of ACTN2, MYH1, MYH2, MYH7, and NEB in Pan-cancers. (**F**) represented the expression levels of ACTN2, MYH1, MYH2, MYH7, and NEB in HPV-positive and -negative HNSC samples. ACC, Adrenocortical carcinoma; BLCA, Bladder Urothelial Carcinoma; BRCA, Breast invasive carcinoma; CESC, Cervical squamous cell carcinoma and endocervical adenocarcinoma; CHOL, Cholangiocarcinoma; COAD, Colon adenocarcinoma; COAD, Colon adenocarcinoma; READ, Rectum adenocarcinoma Esophageal carcinoma; DLBC, Lymphoid Neoplasm Diffuse Large B-cell Lymphoma; ESCA, Esophageal carcinoma; FPPP, FFPE Pilot Phase II; GBM, Glioblastoma multiforme; GBMLGG, Glioma; HNSC, Head and Neck squamous cell carcinoma; KICH, Kidney Chromophobe; KIRC, Kidney renal clear cell carcinoma; KIRP, Kidney renal papillary cell carcinoma; LAML, Acute Myeloid Leukemia; LGG, Brain Lower Grade Glioma; LIHC, Liver hepatocellular carcinoma; LUAD, Lung adenocarcinoma; LUSC, Lung squamous cell carcinoma; MESO, Mesothelioma; OV, Ovarian serous cystadenocarcinoma; PAAD, Pancreatic adenocarcinoma; PCPG, Pheochromocytoma and Paraganglioma; PRAD, Prostate adenocarcinoma; READ, Rectum adenocarcinoma; SARC, Sarcoma; SKCM, Skin Cutaneous Melanoma; STAD, Stomach adenocarcinoma; STES, Stomach and Esophageal carcinoma; TGCT, Testicular Germ Cell Tumors; THCA, Thyroid carcinoma; THYM, Thymoma; UCEC, Uterine Corpus Endometrial Carcinoma; UCS, Uterine Carcinosarcoma; UVM, Uveal Melanoma; AML, Acute Myeloid Leukemia; CCSK, Clear Cell Sarcoma of the Kidney; NBL, Neuroblastoma; OS, Osteosarcoma; RT, Rhabdoid Tumor; WT, High-Risk Wilms Tumor.

Then, we evaluated their expression in other cancers (Fig. 6A–E). ACTN2 was significantly downregulated in bladder urothelial carcinoma (BLCA), kidney chromophobe (KICH), kidney renal papillary cell carcinoma (KIRP), lung adenocarcinoma (LUAD), lung squamous cell carcinoma (LUSC), prostate adenocarcinoma (PRAD), stomach adenocarcinoma (STAD), thyroid carcinoma (THCA), and uterine corpus endometrial carcinoma (UCEC) (*P* < 0.001); up-regulated in invasive breast carcinoma (BRCA), cholangiocarcinoma (CHOL), and colon adenocarcinoma (COAD). We can observe that although MYH1, MYH2, and MYH7 were dysregulated in some of the cancers, their expression levels are very low, except for HNSC. Therefore, MYH1, MYH2, and MYH7 can be considered specific HNSC biomarkers. As for NEB, it was significantly downregulated in BRCA, HNSC, KICH, KIRP, and THCA; up-regulated in BLCA, CHOL, COAD, esophageal carcinoma (ESCA), liver hepatocellular carcinoma (LIHC), LUAD, LUSC, rectum adenocarcinoma (READ), stomach adenocarcinoma (STAD), and UCEC (*P* < 0.05; *P* < 0.01; *P* < 0.001). Moreover, we evaluated the expression levels of ACTN2, MYH1, MYH2, MYH7, and NEB in HPV-positive and -negative HNSC samples. Their expression levels showed no significant differences in HPV-positive and negative HNSC samples (Fig. 6F).

### Immune cell infiltration correlation analysis

**Figure 7:**
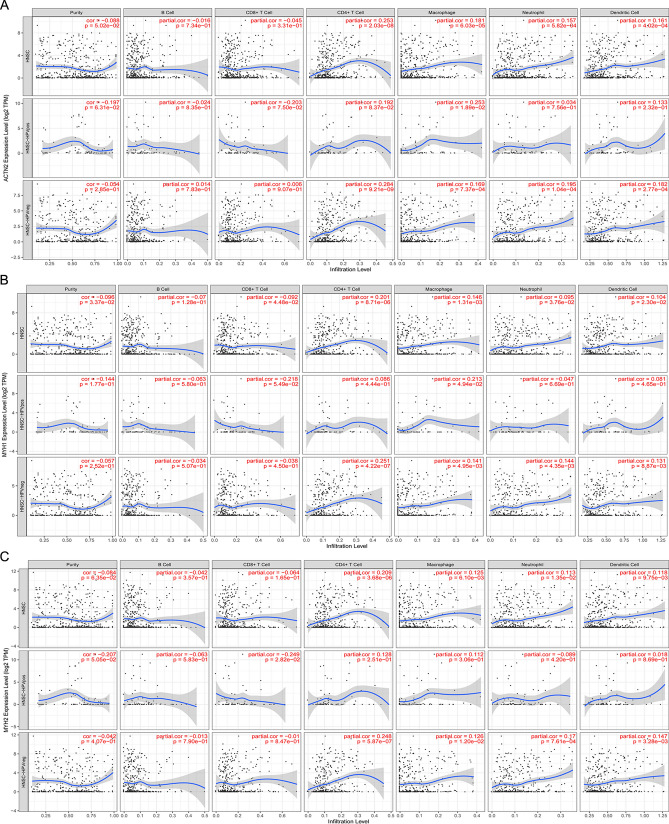

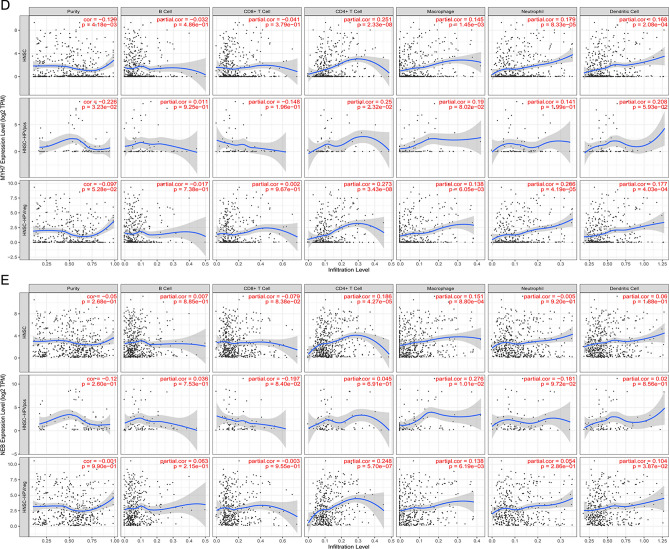
The immune cell infiltration correlation analysis of ACTN2 (**A**), MYH1 (**B**), MYH2 (**C**), MYH7 (**D**), and NEB (**E**) in immune cells. All the immune infiltration correlation analysis was performed in all HNSC samples, HPV-positive, and -negative HNSC samples.

The correlation between key DEGs and immune infiltrate cells in total HNSC, HNSC-positive, and HNSC-negative samples was evaluated. As for total HNSC samples, we found that ACTN2, MYH1, MYH2, and MYH7 were negatively correlated with B cell, CD8+T cell, and positively correlated with CD4+T cell, macrophage, neutrophil, and dendritic cell (Fig. 7A–E). All five DEGs have a significant positive correlation with CD4+T cells and macrophages.

ACTN2 had positive correlation with CD4+T cell (partial correlation = 0.253; *P* = 2.03e−08), macrophage (partial correlation = 0.181; *P* = 6.03e−05), neutrophil (partial correlation = 0.157; *P* = 5.82e-04), and dendritic cell (partial correlation = 0.161; P = 4.02e−04) in HNSC samples. It showed an opposed correlation in HNSC-HPV positive and HNSC-HPV negative in immune cell infiltration of B cell and CD8+T cell, but not significant (Fig. 7A). Therefore, the HPV infection had no significant influence on immune cell infiltration of ACTN2.

MYH1 showed positive correlation with CD4+T cell (partial correlation = 0.201; P = 8.71e−06), macrophage (partial correlation = 0.146; *P* = 1.31e−03), neutrophil (partial correlation = 0.095; *P* = 3.76e−02), and dendritic cell (partial correlation = 0.104; *P* = 2.30e−02; Fig. 7B). The MYH1 showed a consistent correlation in the immune cells of HNSC-HPV positive and negative samples, except in neutrophils. Although MYH1 in HNSC-HPV positive sample is negatively correlated with neutrophils, but is not significant.

As for MYH2, MYH7, and NEB were significantly positively correlated with CD4+T cell (*P* < 0.001; Fig. 7C–E). They showed consistent correlation in the immune cells among the total HNSC, HNSC-HPV positive and negative samples; Some slight correlation opposition was observed in HNSC-HPV positive or negative with total HNSC, but not significant. Therefore, we concluded that no significant differences were identified in HNSC-HPV positive or negative with total HNSC in terms of immune cell infiltration of ACTN2, MYH1, MYH2, MYH7, and NEB.

## Discussion

HNSC, a dreadful opponent, has become one of the world's most deadly diseases. Treatment for HNSC is costly, has a long cycle, and is not affordable. Therefore, the precise and early prediction of HNSC outcomes is critical for therapy options and prognosis improvement. Our present study focused on gene mutation and dysregulation expression in HNSC, expecting to identify essential biomarkers for HNSC.

In the present study, we explored the landscape of mutation and dysregulation in HNSC patients, showing that TP53, TTN, FAT1, CDKN2A, and MUC16 were the most predominant mutated genes. The mutation identification was consistent with Jiang et al.^14^. Among human genes, TP53 is a critical tumor suppressor gene, with low expression in normal cells and high expression in malignant tumors, regulating cell proliferation, apoptosis, angiogenesis, and DNA repair^15^. Mutated TP53 was found in various cancers and is correlated with reduced O.S. Furthermore; it showed that HNSC patients with TP53 mutations have a bleak prognosis than TP53-wildtype HNSC^16^. Our present study also found that mutated TP53 is observed in HNSC.

Then, the dysregulated genes were identified, with 187 DEGs having more mutation sites. We conducted the PPI analysis on these DEGs and identified two modules. We further analyzed these DEGs in these modules by GO-BP and KEGG analyses. The DEGs in module A are involved in the PI3K-Akt signaling pathway, a classic intracellular signaling pathway regulating cell apoptosis, proan essential pathway that regulates cell apoptosis, prognosis, and metastasis in HNSC^17–19^. The DEGs in module B participated in the cGMP-PKG signaling pathway. In addition, Tuttle et al.^20^ demonstrated that the cGMP-PKG signaling pathway is one of the therapeutic targets of HNSC.

The DEGs of ACTN2, MYH1, MYH2, MYH7, and NEB in module B were significantly associated with O.S. in HNSC. ACTN2 is a member of the spectrin gene superfamily that includes varying groups of cytoskeletal proteins. In breast cancer patients, mutated ACTN2 was related to invasive ductal carcinoma and suggested a worse O.S. than ductal carcinoma in situ^21^. Sun et al.^22^ demonstrated that ACTN2 is one of the hub genes selected by bioinformatics methods in PTEN mutation prostate cancer. Xu et al.^23^ revealed that negative ACTN2 expression contributed to a better O.S. in HNSC patients, consistent with our present study. Therefore, ACTN2 might be an essential biomarker for predicting O.S. of HNSC, which might be employed in clinical.

Through pan-cancer analysis, we found that MYH1, MYH2, and MYH7 are dysregulated in cancers, including HNSC. However, compared with HNSC, they were lower expressed in other cancers, revealing that these three genes can be considered single HNSC biomarkers. So far, different mutations in multiple members of the MYH gene family are associated with human hereditary diseases^24^. Among them, MYH2 mutations can cause a class of skeletal muscle diseases characterized by ophthalmoplegia. MYH7 mutations can cause skeletal muscle diseases, including myosin deposition myopathy, and Distal Laing myopathy is also closely related to hypertrophic cardiomyopathy. However, MYH1, MYH2, and MYH7 functions in HNSC are rarely mentioned. Further study into the interaction of the MYH gene family and HHSC appears to be an intriguing research topic.

As we all know, HPV infection is the most common causative factor for HNSC. Considering the significant influence of HPV on HNSC, some molecular research on HNSC have divided the samples into HNSC-HPV positive and negative groups. For example, Zhang et al.^25^ found that Dedicator of cytokinesis 8 (DOCK8) is identified as one prognosis biomarker associated with immune infiltration HPV-positive HNSC. Our present study identified five prognosis DEGs that are significantly related to the O.S. in HNSC. To determine whether the HPV infection had significant influences on the results. We further analyzed their expression in HPV-positive and -negative HNSCs, and no significant differences were observed. Moreover, we also tested the immune cell infiltration of DEGs in both HPV-positive and -negative HNSCs. Although studies have demonstrated that HPV influences the immune infiltration situation^26^, it is not observed in ACTN2, MYH1, MYH2, MYH7, and NEB.As genes highly expressed in the tumor cells are expected to affect tumor purity positively, the association between the expression of prognosis DEGs and six immune infiltrates was evaluated. We found that all five DEGs have a significant positive correlation with CD4+T cells and macrophages. Therefore, we speculate that genetic mutations and differential expression of ACTN2, MYH1, MYH2, MYH7, and NEB genes in HNSC cells may be important drivers of CD4+T cell and macrophage infiltration.

## Conclusions

In our present study, we identified the biomarker of HNSC in terms of diagnosis and prognosis. TP53 was found as the most mutated gene in HNSC. ACTN2, MYH1, MYH2, MYH7, and NEB genes were significantly associated with poor prognosis. Moreover, MYH1, MYH2, and MYH7 were expected to be the specific diagnostic and prognostic biomarkers molecular biomarkers of HNSC.

## Methods

### Data collection

The clinical data and gene expression profiles of HNSC patients were downloaded from the UCSC Xena database (https://xenabrowser.net/datapages/). As calculated, a total of 506 tumors and 44 adjacent HNSC samples were included in our present study. The gene expression levels of examples were provided in Supplementary File 2. The somatic mutation data was from the TCGA database by Varscan^27^.

### Screening of high-frequency mutation genes

The maftools^28^ was used to summarize the HNSC mutation information downloaded from the TCGA official website and screen the high-frequency mutation genes. The top 25 high-frequency genes with the mutation frequency were exhibited with a waterfall chart.

### Differentially expressed gene (DEG) identification

The DEGs in tumor tissues were identified according to the FPKM value by limma package^29^ (Version 3.10.3), using *P*-value < 0.05 and log_2_ fold change (F.C.) > 2 as thresholds. The Pheatmap R package was employed for drawing the heatmap and volcano.

Then, the common DEGs with high-frequency mutation genes that exhibit a mutation frequency of more than 1% were retained for further study.

### Protein–protein interaction (PPI) and module analyses

The PPI analysis was performed on all the common DEGs using STRING^30^ (Version: 10.0, http://www.string-db.org/) database, with a 0.4 (medium confidence) parameter. The network was constructed by Cytoscape (version: 3.2.0)^31^. The most significant clustered modules in the PPI network were analyzed using the Cytoscape plug-in MCODE^32^ (Version 1.4.2, http://apps.cytoscape.org/apps/MCODE) method, with a threshold of score ≥ 10.

### Functional analysis

The biological process (B.P.) of Gene Ontology (G.O.) and the Kyoto Encyclopedia of Genes and Genomes (KEGG) analyses on DEGs were performed by clusterProfiler^33^, with adjusted *P*-value < 0.05 and gene number count ≥ 2. The top ten G.O. terms and KEGG pathways were visualized.

### Key DEG expression validation and survival curve analysis

The key DEGs in the module were subjected to expression verification and survival analysis by GEPIA^34^. Survival analysis was grouped by median data and performed using OS survival information. The pan-cancer expression levels of key DEGs significantly associated with prognosis were assessed using UALCAN (http://ualcan.path.uab.edu/index.html). The expression levels of key DEGs in HNSC-positive and HNSC-negative samples were evaluated.

### Immune cell infiltration analysis

As for the immune cell infiltration analysis, the HNSC samples were divided into HNSC-positive and HNSC-negative. Then, the infiltration of the key DEGs in six immune cells (B cells, CD^4+^ T cells, CD^8+^ T cells, neutrophils, macrophages, and dendritic cells) were identified through the TIMER database.

### Ethics approval and consent to participate

UCSC Xena and TCGA belongs to public databases. The patients involved in the database have obtained ethical approval. All methods were carried out in accordance with relevant guidelines and regulations.

## Supplementary Information

Supplementary Information 1.

Supplementary Information 2.

## Data Availability

The RNA-Seq and mutation data re-analyzed during the current study are available in the TCGA database with the links of https://xenabrowser.net/datapages/?dataset=TCGA-HNSC.htseq_fpkm.tsv&host=https%3A%2F%2Fgdc.xenahubs.net and https://xenabrowser.net/datapages/?dataset=TCGA-HNSC.varscan2_snv.tsv&host=https%3A%2F%2Fgdc.xenahubs.net.
